# Commentary: Stimulation of the Posterior Cortical-Hippocampal Network Enhances Precision of Memory Recollection

**DOI:** 10.3389/fpsyg.2017.00899

**Published:** 2017-05-30

**Authors:** Ting Liu, Mingchen Fu, Tifei Yuan, Dong-Wu Xu

**Affiliations:** ^1^School of Psychology, Nanjing Normal UniversityNanjing, China; ^2^School of Education Science, Nanjing Normal UniversityNanjing, China; ^3^Faculty of Education, University of Hong KongHong Kong, Hong Kong; ^4^School of Mental Health, Wenzhou Medical UniversityWenzhou, China

**Keywords:** memory, TMS, cognition, memory enhancement, tDCS

Memory is critical to our daily life. Various strategies have been employed to promote cognitive performance, such as pharmacological tools, physical exercise, and cognitive training (Stern and Alberini, 2013; Kelly, 2015). In recent years, non-invasive neuromodulation techniques [e.g., transcranial magnetic stimulation (TMS) and transcranial direct current stimulation (tDCS); Sparing and Mottaghy, 2008; Hansen, 2014; Rose et al., 2016; Voarino et al., 2016] are demonstrating prospective future in memory retrieval and enhancement.

Episodic memory refers to the memory of specific events taken place in the past, which relies on the interactions between hippocampus and other brain regions (Ranganath and Ritchey, 2012). Among various brain regions that mediate different functions, the hippocampal posterior-medial (HPM) network is specifically associated with the recall of highly precise contextual and spatial information (Nadel et al., 2013). In a previous study, Rose et al. demonstrated that TMS was effective for improving memory performance (Rose et al., 2016). Another study utilized TMS to stimulate the HPM network and found that TMS could improve the recollection of paired-associate cued items and increase functional connectivity within the HPM network (Wang et al., 2014). However, it was still unknown if this intervention would improve memory precision.

The recent study published in Current Biology by Nilakantan et al. explored for the first time the causal role of the HPM network in recollection precision (Nilakantan et al., 2017). Recollection precision refers to the memories that require the recall on details of the targeted objects, which is distinguished from general recollection in memory tasks. In this study, the authors employed an object-location memory task and participants were asked to memorize the object-locations as accurately as possible. Based on high functional connectivity between the lateral-parietal cortex and hippocampus (Kahn et al., 2008), the researchers chose the lateral-parietal cortex as the stimulation site to target HPM network as previous study (Wang et al., 2014). On the second day, participants were asked to perform the task, followed by 5-days' continuous repetitive TMS (rTMS) and another object-locations task with electroencephalogram/event-related potentials (EEG/ERP) recorded. The results demonstrated that HPM targeting TMS stimulation could reduce the ERP responses to recollection, associated with significant improve in recalling precision.

Nilakantan et al. used TMS to change episodic memory precision and extended previous lesion-based studies for human brain network involvement in memory. Besides, TMS can be also applied to modulate other forms of memory. A study showed that rhythmic TMS could enhance auditory working memory performance (Albouy et al., 2017). Rademaker et al. also proved that a visual working memory precision-enhancing effect could be found at visual cortex (Rademaker et al., 2017). Therefore, TMS may have great potential value in human memory system in the future.

With the power of image-guided TMS, researchers can activate a specific circuit and explore its effect in human brain. Previous studies have explored the effect of TMS on human cognition (Luber and Lisanby, 2014). TMS was adopted to activate latent working memory with a relatively short temporal efficiency (Rose et al., 2016). In present study, 5-days' continuous TMS treatment enhance memory for at least 24 hours. It can be implied from the study that continuous rTMS is considerably more effective for enhancing memories than short and sporadic stimulations.

There were still some limitations of the present study. First, the mechanisms underlying the TMS-induced enhancement of HPM network activity are unclear. Future studies are required to confirm the importance of structure modification or functional changes in these neural circuits. Furthermore, most TMS coils only target the cortical layers in the brain and activate subcortical structures indirectly. In the future, it may be possible to develop deep brain stimulation-inspired TMS stimulation with more focused TMS coils or deep TMS coils. Last but not least, the stimulation parameters could be optimized to further enhance memory.

In the future, it will be interesting to understand if interventions in the HPM network could as well reduce certain memory precisely, and erase unwanted memory as the case for post-traumatic stress disorder (PTSD). In fact, pairing stimulation could result in bi-directional plasticity with varied temporal interval (Koch et al., 2013), and such approach would allow for the “un-learning” of “dark memories,” including fear and addiction.

## Author contributions

All authors designed the study, wrote the manuscript together, and approved the final version of the manuscript. TL and MF contributed equally to this work.

### Conflict of interest statement

The authors declare that the research was conducted in the absence of any commercial or financial relationships that could be construed as a potential conflict of interest.
